# Reversible proton-switchable fluorescence controlled by conjugation effect in an organically-functionalized polyoxometalate

**DOI:** 10.1038/srep27861

**Published:** 2016-06-20

**Authors:** Chunlin Lv, Kun Chen, Junjie Hu, Jin Zhang, Rao Naumaan Nasim Khan, Yongge Wei

**Affiliations:** 1 Department of Chemistry, Tsinghua University, Beijing 100084, China; 2 Solid Waste and Chemicals Management Center, Ministry of Environmental Protection, Beijing 100029, China; 3 State Key Laboratory of Natural and Biomimetic Drugs, Peking University, Beijing 100191, China

## Abstract

A novel monosubstituted organoimido hexamolybdate containing 6-nitroquinoline moiety has been successfully synthesized. This organically-functionalized polyoxometalate exhibits proton-induced switchable fluorescence property in aqueous acetonitrile solution at room temperature. Experimental and theoretical investigations of this reversible “on” and “off” switching mechanism have been carried out, and it is found that the protonation and deprotonation at the heterocyclic nitrogen atom within quinoline fragment leads to the breaking and reformation of the conjugation through strong d-π interaction between the hexamolybdate anionic cluster and the quinoline moiety, resulting in “on” and “off” luminescence signal.

Molecular switches constitute a variety of reporter molecules able to show response to external stimuli by the reversible molecular structural change such as *cis-trans* isomerization, ring-closing/ring-opening, molecular proton-transfer processes, behavior of assembly and so on^1^,^2^,^3^. As an efficient and highly sensitive method, fluorescent molecular switches have attracted substantial research interest in the past decades^4^,^5^,^6^,^7^,^8^,^9^. In particular, proton sensors which can indicate the presence of protons are of great use in the field of detectors response to environmental changes, memories or logic gates in nanotechnology, and especially life sciences^10^,^11^. Up to date, though numerous fluorescent systems exhibiting reversible response to protons have been reported, the new challenge of analysis constantly needs to develop more proton-responsive fluorescent sensors. Therefore, the development of molecular switches having fluorescent response to external stimuli represents a continuous research topic.

In this sense, polyoxometalates (POMs) which are a rich class of metal oxide anionic clusters with diversity of structure and unique photonic, electronic, and magnetic properties^12^,^13^,^14^,^15^,^16^ are good candidates for fabricating multifunctional fluorescent materials. Previously, one well understood synthetic method was to incorporate lanthanide ions, mainly Eu^3+^ and Tb^3+^, into POMs^17^,^18^,^19^,^20^,^21^,^22^. Alternatively, the introduction of organic fluorophore may be an efficient synthetic strategy and have been attracted much attention in the past decade^23^,^24^,^25^,^26^,^27^,^28^,^29^,^30^,^31^,^32^. Especially, the organic functionalization of POMs *via* covalent linkage to form organic-inorganic hybrid fluorescent materials is a very attractive and challenging research area. Much pioneer work has been done by Neumann and Peng^27^,^28^,^29^,^30^, however it is found that POM clusters exhibit efficient fluorescence quenching when the inorganic clusters directly linked to the organic conjugated polymers or moieties through Mo-N triple bond^28^,^29^,^30^. Recently, Liu and Wei and co-workers reported that surfactant functionalized hexavanadates with proton or sodium ions as the counter-ions have fluorescent property^31^. Meanwhile, Liu and Hill and co-workers also found this photoluminescence in inorganic-organic hybrid hexavanadate clusters containing pyrene moiety^32^. However, to the best of our knowledge, POMs-based single-molecular switches displaying reversible “on” and “off” photoluminescence response to protons have not been reported yet.

In this paper, we report the synthesis of a novel organoimido hexamolybdate containing 6-nitroquinoline moiety, which exhibits reversible stimuli-response to protons. Furthermore, the mechanism of this reversible fluorescent behavior based on electron delocalization breaking and reforming is also investigated in detail.

## Results

### Synthesis and characterization

A reaction of the hexamolybdate salt ((Bu_4_N)_2_[Mo_6_O_19_]) with 5-amino-6-nitro quinoline in the ratio of 1:1 using the well-established DCC (N,N′-dicyclohexylcarbodiimide) protocol^33^ can conveniently give rise to the corresponding monosubstituted organoimido derivative, (Bu_4_N)_2_[Mo_6_O_18_(NC_9_H_5_N-NO_2_)] (Mo_6_-Q-NO_2_), which is crystallized as orange lamellar-like crystals when diethyl ether diffuses into their solution of acetonitrile. The IR spectrum of compound Mo_6_-Q-NO_2_ exhibits similar characteristic features to those of the reported monosubstituted organoimido hexamolybdates (Supplementary Fig. S1). In addition, the ESI-MS of this compound shows clearly resolved peaks at 525.6 and 1293.9 which correspond to [Mo_6_O_18_(NC_9_H_5_N-NO_2_)]^2^^−^ (calcd. 525.4) and (Bu_4_N)[Mo_6_O_18_(NC_9_H_5_N-NO_2_)]^−^ (calcd. 1293.3) (Supplementary Fig. S2), respectively.

**Figure 1:**
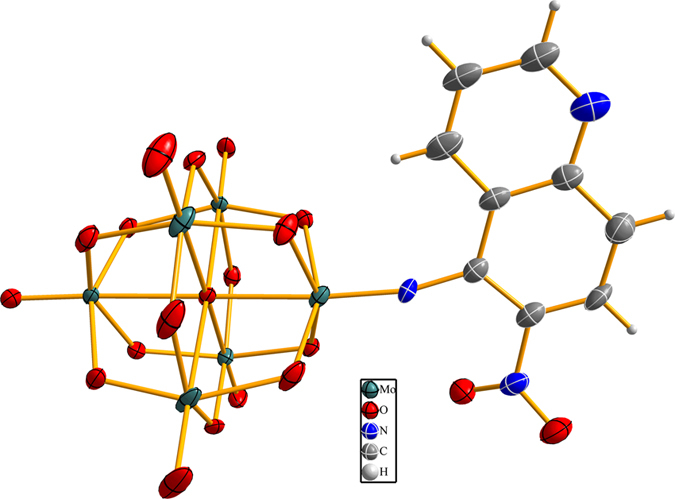
ORTEP drawing of the cluster anion within Mo_6_-Q-NO_2_. Mo teal, O red, N blue, C gray, H white. (25% probability ellipsoids).

Furthermore, single-crystal X-ray diffraction investigation has also been performed. It is found that an asymmetric unit contains one independent cluster anion and two counter tetrabutylammonium cations as well as a half of acetonitrile molecule and one water molecule. The ORTEP drawings of the anionic cluster within compound Mo_6_-Q-NO_2_ is presented in Fig. 1. As shown in Fig. 1, compound Mo_6_-Q-NO_2_ displays the typical structural features as those of monofunctionalized hexamolybdates: one terminal oxo atom (O_t_) of the hexamolybdate cluster has been replaced by an organoimido ligand. In addition, the formed Mo–N bond is found to be 1.730 Ǻ, and the nearly linear Mo–N–C angle is refined as 167.1° (Supplementary Tables S1 and S2), exhibiting the typical triple bond features^34^.

### Photo-physical property

**Figure 2:**
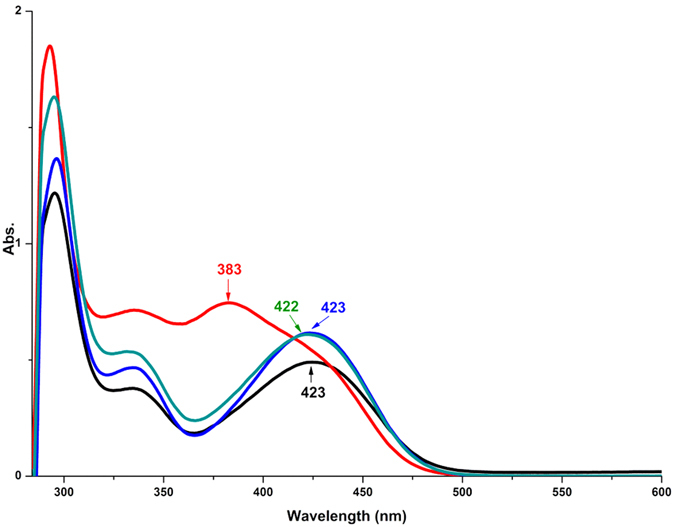
UV-Vis absorption spectra of Mo_6_-Q-NO_2_ in mixed solutions. Mo_6_-Q-NO_2_ in mixed acetonitrile and water solution (green line), in mixed acetonitrile and water solution after adding dilute HCl solution (red line), in mixed acetonitrile and water solution after adding dilute NaOH solution (blue line), as well as in the acidic solution neutralized with dilute NaOH solution (black line).

The UV-Vis spectrum of compound Mo_6_-Q-NO_2_ is shown in Fig. 2. The lowest-energy electronic transition (LET) absorption band in 50% aqueous acetonitrile solution locates at 423 nm, which exhibits obviously bathochromic shift compared to the parent hexamolybdate due to the quinoline aromatic ring conjugated with the hexamolybdate framework through the strong d-π interaction^35^. To assess the effect of acid and base, absorption changes of Mo_6_-Q-NO_2_ in the presence of 10 equivalents of H^+^ and OH^−^ in 50% aqueous acetonitrile solution have been determined respectively. It is found that the LET absorption band has no obviously change in the presence of 10 equivalents of OH^−^, while the LET absorption band has significant hypsochromic shift from 423 nm to 383 nm in the presence of 10 equivalents of H^+^. Interestingly, upon further addition of 0.5 mL dilute NaOH solution (10 equivalents) into the acidic solution to neutralize the HCl, the LET absorption band migrates back to 423 nm. Note that the decreased intensity of absorption peaks attributes to the dilution effect of the addition of 0.5 mL dilute NaOH solution. This reversible change of LET absorption band not only indicates that Mo_6_-Q-NO_2_ is stable after these treatments but also implies that a corresponding reversible change of conjugate delocalization system within compound Mo_6_-Q-NO_2_ takes place.

### Theoretical investigation of the protonated structure

**Figure 3:**
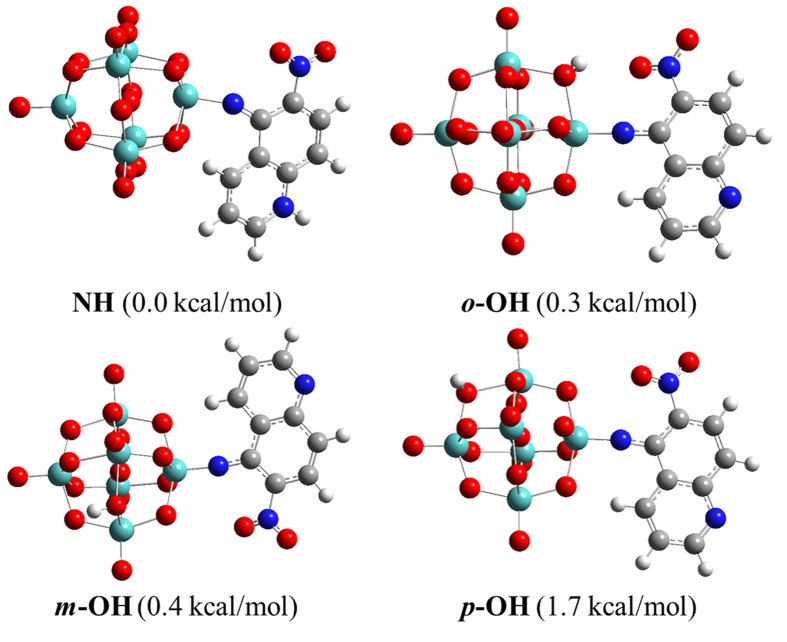
Optimized structures of Mo_6_-Q-NO_2_-H.

To explore the cause of reversible change of LET absorption band, we firstly need to determine the protonation site. Previous studies have pointed out that bridge oxygen (O_b_) is a preferable binding site to proton as compared to terminal oxygen^36^,^37^,^38^,^39^. Therefore, within Mo_6_-Q-NO_2_ there are four possible protonation sites which are bridge oxygen atoms bound to the molybdate atom at the *trans* site of the imido molybdate atom (*p*-O_b_), bridge oxygen atoms only bound to the molybdate atom at the *cis* site of the imido molybdate atom (*m*-O_b_), and bridge oxygen atoms bound to imido molybdate atom (*o*-O_b_) as well as the heterocyclic nitrogen atom. The corresponding structures are defined as *p*-OH, *m*-OH, *o*-OH and NH, respectively. By the optimization of their structures using DFT-B3LYP method^40^,^41^, shown in Fig. 3, it was found that the structure corresponding to the protonation at the heterocyclic nitrogen atom is the most stable isomer. Based on this result, we can explain the reversible change of absorption band from the corresponding change of structure. The protonation at the heterocyclic nitrogen atom leads to the Mulliken charge of the hydrogen bound to the 4 site carbon in quinoline moiety increased from 0.362 to 0.555, resulting in much stronger hydrogen bonding between 4-H and the bridge atom O15. Combination of the effect of hindrance of 6-nitro, in addition, the angle of Mo-N-C decreased to 150.5°. Simultaneously, the bond length of Mo-N increased to 1.851 Ǻ while that of N-C decreased to 1.315 Ǻ (Supplementary Fig. S3). All these changes of structure indicate that the Mo-N bond exhibits much more like single bond, largely decreasing the d-π interaction between inorganic hexamolybdate cluster and organic quinoline aromatic ring. Therefore, the absorption band in UV-Vis spectrum shifts to 383 nm after protonation.

### Reversible proton-switchable fluorescence

**Figure 4:**
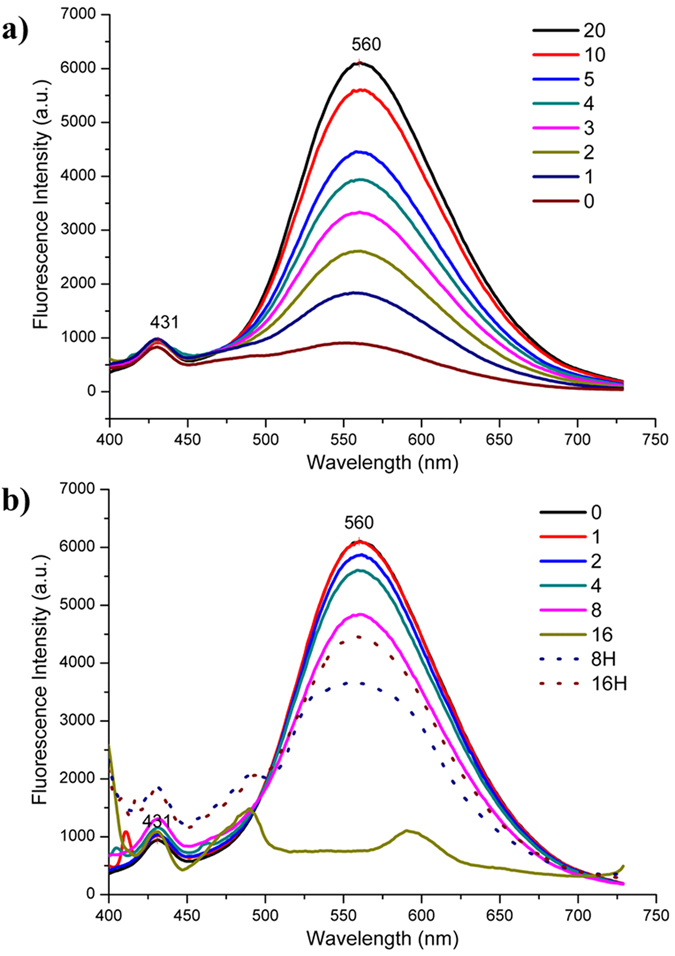
Fluorescence emission spectra of Mo_6_-Q-NO_2_. (**a**) The increasing fluorescence emission spectra of Mo_6_-Q-NO_2_ excited at 377 nm in acetonitrile (2.0 × 10^−4^ mol/L) with adding 0~20 equimolar dilute HCl; (**b**) The decreasing fluorescence emission spectra of Mo_6_-Q-NO_2_ in acetonitrile with the neutralization of the acidic solution by adding 0~16 equimolar dilute NaOH (solid line) and re-increasing fluorescence emission spectra with re-adding 8 and 16 equimolar dilute HCl (dotted line).

In previous investigations, it was found that the Mo-N triple bond linking POM clusters with conjugated aromatic moiety displayed significant fluorescence quenching because of the photo-induced electron transfer from the fluorophore to the clusters^27^,^28^,^29^,^30^. Likewise, compound Mo_6_-Q-NO_2_ also exhibits no fluorescence. However, compound Mo_6_-Q-NO_2_ clearly displays proton responsive “on” fluorescence at 560 nm with excitation wavelength of 377 nm. As shown in Fig. 4a, upon gradual addition of dilute HCl (0~20 equimolar) to the 50% aqueous acetonitrile solution of Mo_6_-Q-NO_2_, the intensity of the emission at 560 nm increased gradually. While upon further gradual addition of dilute NaOH (0~16 equimolar) to the acidic solution, the emission intensity exhibited gradual decreasing until the fluorescence almost “off”, (presented in Fig. 4b). To verify this reversible “on” and “off” fluorescence originated from the presence and absence of H^+^, further gradual addition of 8 and 16 equimolar dilute HCl have been performed, and the recovered fluorescence is observed. A control experiment confirmed that this proton-switchable fluorescence was due to Mo_6_-Q-NO_2_, as the organic ligand 5-amino-6-nitroquinoline showed almost no fluorescence with excitation wavelength of 377 nm (Supplementary Fig. S4). It is noteworthy that this is a first example of organoimido derivative of POMs with significant reversible proton-switchable fluorescent property.

## Discussion

**Figure 5:**
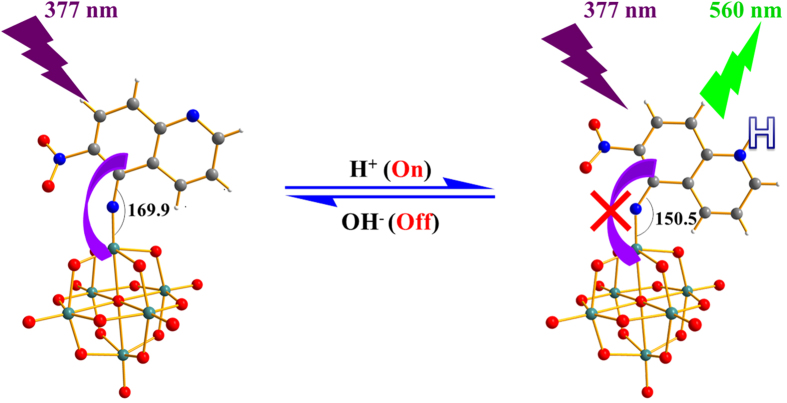
Schematic illustration of switchable fluorescence response to proton. Proposed mechanism for Mo_6_-Q-NO_2_ displaying single-molecule switchable fluorescence response to proton.

In light of the structural analysis as above, fluorescence enhancement upon gradual addition of dilute HCl into the 50% aqueous acetonitrile solution of Mo_6_-Q-NO_2_ is due to the protonation at the heterocyclic nitrogen atom leading to the Mo-N changing from triple bond to single bond, resulting in the effective channel of photo-induced electron transfer from the organic 6-nitroquinoline moiety to the hexamolybdate cluster that is blocked. Contrarily, the quenching of fluorescence upon addition of NaOH into this system attributes to subsequent deprotonation of the heterocyclic nitrogen atom, regenerating the effective photo-induced electron transfer channel, illustrated in Fig. 5.

In summary, we have successfully synthesized a novel mono-substituted organoimido hexamolybdate containing isoquinoline moiety which, as a first example of organoimido- POMs-based fluorescent single-molecule switch, exhibits proton-induced switchable fluorescence property in aqueous acetonitrile solution at room temperature. Moreover, experimental and theoretical investigations of reversible “on” and “off” switching mechanism have been carried out, and it is found that the protonation and deprotonation at the heterocyclic nitrogen atom within quinoline fragment leads to the breaking and reformation of the conjugation through strong d-π interaction between the hexamolybdate anionic cluster and the quinoline moiety, resulting in “on” and “off” luminescence signal. This proton-responsive switchable fluorescent activity provides a potential application in sensors using organic functionalized polyoxometalates. More importantly, this work paves a new way to design and synthesize POM-based organic-inorganic hybrid fluorescent single-molecule switches.

## Methods

### Materials

(Bu_4_N)_2_[Mo_6_O_19_] was synthesized according to the improved literature method^42^ and dried before use. 5-amino-6-nitroquinoline was purchased from Alfa Aesar. Acetonitrile was dried by refluxing in the presence of CaH_2_ and was distilled prior to use. N,N′-dicyclohexylcarbodiimide (DCC) and ethyl ether were directly used without further purification.

### Spectroscopic Characterization

Infrared spectra were measured using the method of KBr pellets and recorded on a Perkin-Elmer FTIR spectrometer. The electrospray mass spectra (ESI-MS) were measured on a Bruker Apex IV FTMS Plus ion-trap mass spectrometer, and all experiments were carried out in the negative-ion mode using CH_3_CN as solvent. UV-Vis absorption spectra of Mo_6_-Q-NO_2_ in a mixed solution of acetonitrile and water were recorded on a UN-2100s spectrometer. Fluorescence measurements were recorded in 3 mL quartz cuvettes at room temperature using a Hitachi F-7000 FL Spectrophotometer equipped with a xenon lamp excitation source. All fluorescence spectra were measured at an excitation wavelength of 377 nm.

### Crystallographic structural determinations

A suitable crystal of the compound Mo_6_-Q-NO_2_ was covered with petroleum jelly, mounted onto glass fibers, and transferred directly to a Rigaku RAXIS-SPIDER IP diffractometer. Data collection, data reduction, cell refinement, and experimental absorption correction were performed with the software package of Rigaku RAPID AUTO (Rigaku, 1998, Ver2.30). Structures were solved by direct methods and refined against F^2^ by full matrix least squares. All non-hydrogen atoms, except disordered atoms, were refined anisotropically. Hydrogen atoms were generated geometrically. All calculations were performed using the SHELXTL V. 5.10 program^43^.

### Theoretical calculations

All the calculations presented in this work were carried out with the Gaussian-09 program package^44^. The structures of each stationary point were fully optimized using the B3LYP method, in combination with the LANL2DZ basis set for molybdate atoms and the 6-31G(d) basis set for rest atoms. Vibrational frequencies of each stationary point were calculated at the same level of theory to characterize the nature of the stationary points and give the thermal correction to Gibbs free energy. In addition, to consider the solvent effect, a single point energy calculation was performed by using the integral equation formulism within the polarized continuum model (IEF-PCM)^45^ with the Bondi atomic radii for the united atom topological model at the B3LYP level based on each optimized geometry, with the LANL2DZ basis set for molybdate atoms and the 6–31 + G(d, p) basis set for rest atoms denoted as IEFPCM-B3LYP/BS1. The dielectric constant of acetonitrile is the default value of the G09 program.

### Synthesis of (Bu_4_N)_2_[Mo_6_O_18_(NC_9_H_5_N-NO_2_)] (Mo_6_-Q-NO_2_)

A mixture of (Bu_4_N)_2_[Mo_6_O_19_] (1.36 g, 1.0 mmol), 5-amino-6-nitroquinoline (0.189 g, 1.0 mmol), and DCC (0.23 g, 1.1 mmol) dissolved in 15 mL anhydrous acetonitrile and then heated to reflux at 110 °C for about 20 h. After being cooled to room temperature, the reaction solution was filtrated to remove the white precipitates of 1,3-dicyclohexylurea (DCU) and then the filtrate poured into ether, resulting in precipitates to come out. After the solution changing to clarify, the supernatant liquid was poured off, and the resulting small crystals were compound Mo_6_-Q-NO_2_ (0.476 g, yield: 31%). By diffusion of Et_2_O into their solution in acetonitrile, orange lamellar-like crystals which are suitable for the X-ray diffraction were obtained for compound Mo_6_-Q-NO_2_. IR (KBr): 797, 953, 977, 1152, 1321, 1386, 1420, 1481, 1556, 2873, 2933, 2961 cm^−1^. UV/Vis (MeCN): λ_max_ = 423 nm. ESI-MS(m/z): 1293.9 ([Mo_6_O_18_(NC_9_H_5_N-NO_2_ + TBA]^−^, calcd 1293.3), 525.6 ([Mo_6_O_18_(NC_9_H_5_N-NO_2_]^2−^, calcd 525.4). Crystal data for compound (Mo_6_-Q-NO_2_)(MeCN)_0.5_(H_2_O): C_42_H_80.5_Mo_6_N_5.5_O_21_, M = 1574.26, Monoclinic, space group *P-1* (No. 1), *a* = 12.6462(8), *b* = 12.7095(8), *c* = 19.779(2) Å, *α* = 101.371(7)°, *β* = 102.654(7)°, *γ* = 101.842(6)°, *V* = 2936.6(4) Å^3^, *T* = 103 K, *Z* = 2, Dc = 1.780 g cm^−3^, m = 1.316 mm^−1^, 30329 collected reflections, 11522 independent (R_int_ = 0.0446), GooF = 1.068, Final *R* indices (I ≥ 2σ(I)) *R*_1_ = 0.0647, w*R*_2_ = 0.1260.

## Additional Information

**How to cite this article**: Lv, C. *et al*. Reversible proton-switchable fluorescence controlled by conjugation effect in an organically-functionalized polyoxometalate. *Sci. Rep.* **6**, 27861; doi: 10.1038/srep27861 (2016).

## Supplementary Material

Supplementary Information

Supplementary Dataset 1

Supplementary Dataset 2
