# Supplementary material for: Reversible proton-switchable fluorescence controlled by conjugation effect in an organically-functionalized polyoxometalate
Source: Sci Rep. 2016 Jun 20;6:27861. doi: 10.1038/srep27861 (PMC4913239; doi:10.1038/srep27861)
Supplement: Supplementary Information [file srep27861-s1.doc]

**Supporting Information For**

Reversible Proton-switchable Fluorescence Controlled by Conjugation Effect in an Organically-Functionalized Polyoxometalate

Chunlin Lv^1,+^, Kun Chen^1,+^, Junjie Hu^2^, Jin Zhang^1^, Rao Naumaan Nasim Khan^1^, Yongge Wei^1,3^,*

^1^ Department of Chemistry, Tsinghua University, Beijing 100084, China.

^2^ Solid Waste and Chemicals Management Center, Ministry of Environmental Protection, Beijing 100029, China.

^3^ State Key Laboratory of Natural and Biomimetic Drugs, Peking University, Beijing, 100191, China.

*Authors to whom all correspondence should be addressed. Tel: +86-10-62797852; Fax: +86-10-62797852. Email: yonggewei@mail.tsinghua.edu.cn

^+^ these authors contributed equally to this work


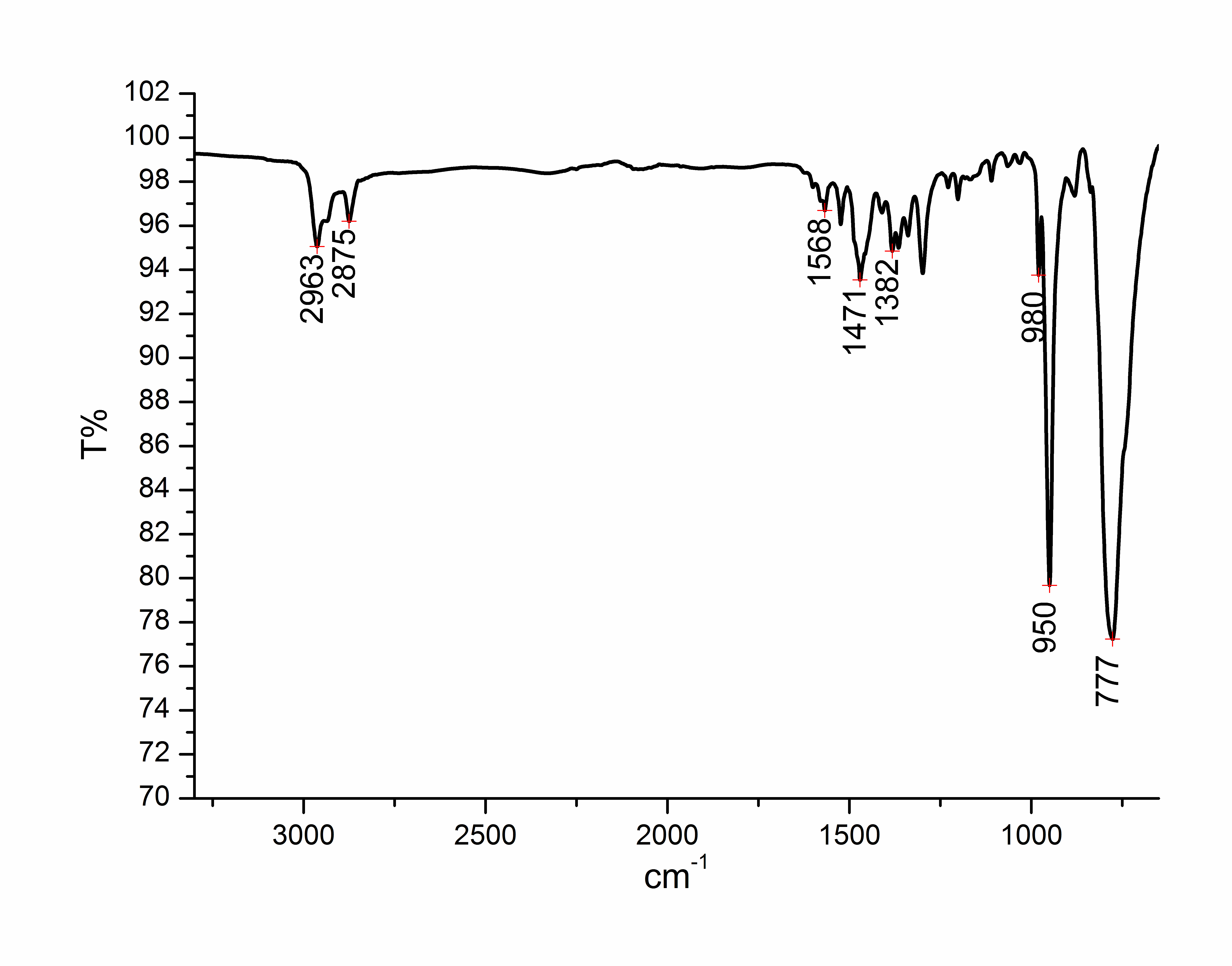


**Figure S1** IR spectrum of compound **Mo_6_-Q-NO_2_**.


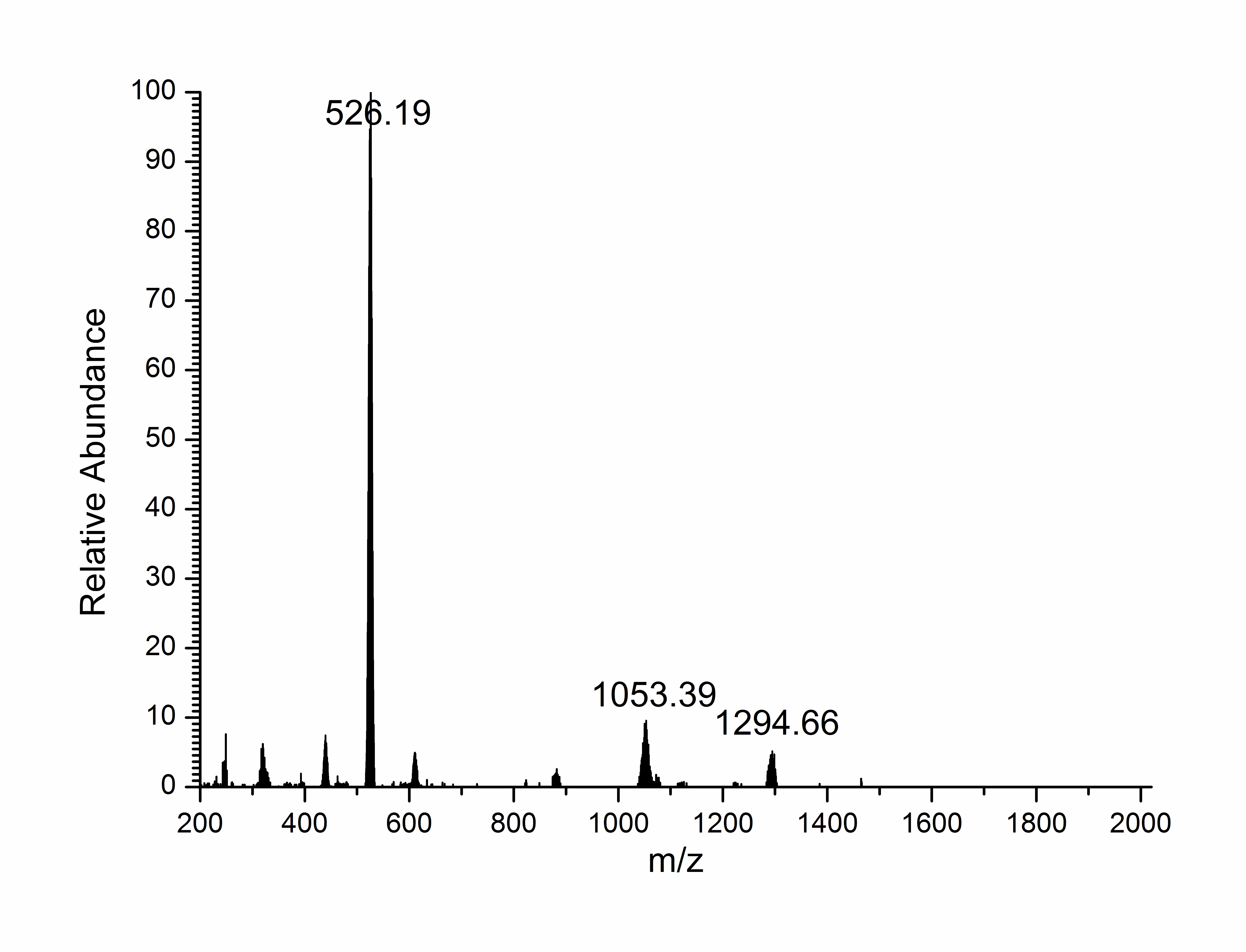


**Figure S2** ESI-MS spectrum of compound **Mo_6_-Q-NO_2_**.


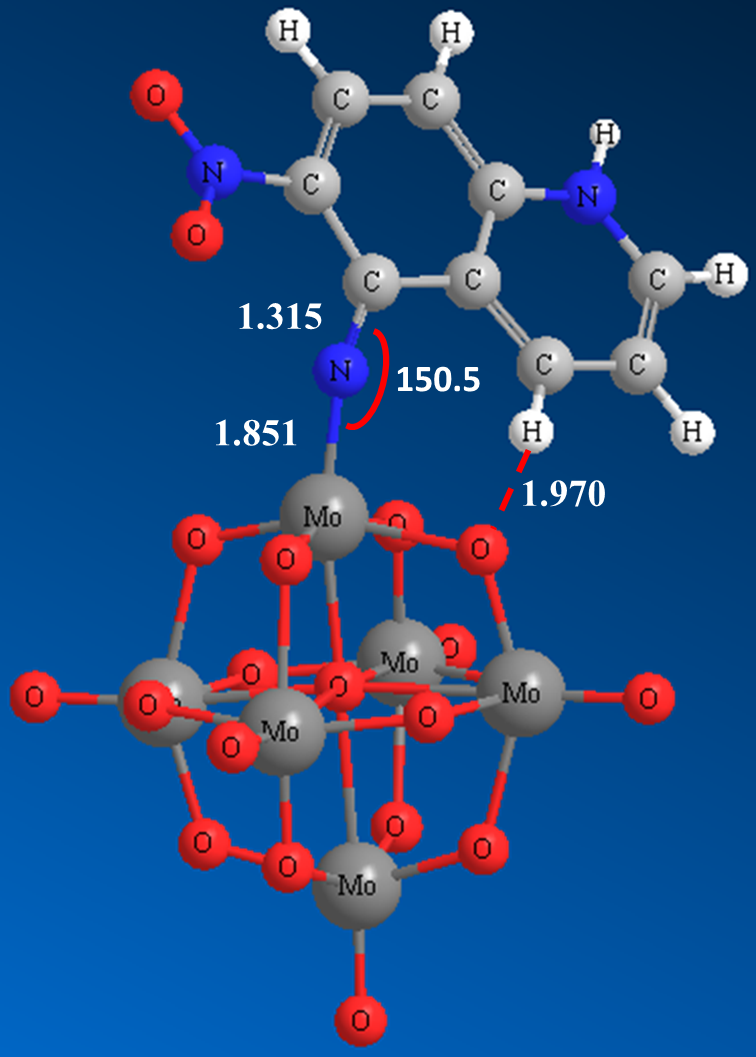


**Figure S3** Optimized structure of **NH** (protonated **Mo_6_-Q-NO_2_** at heterocycle nitrogen atom**)** .

**
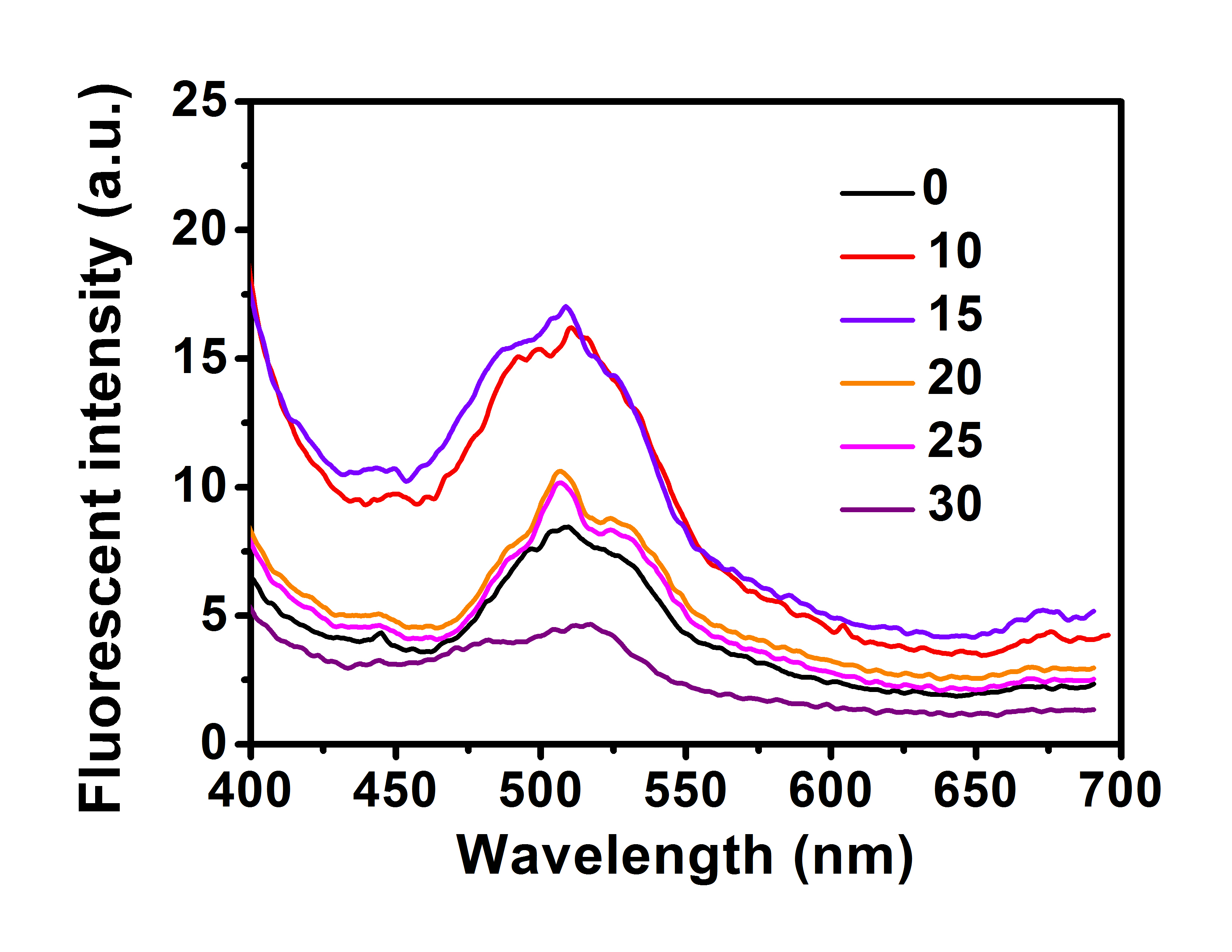
**

**Figure S4** The fluorescent emission spectra of 5-amino-6-nitroquinoline in DMSO (0.2 mM) with adding 0~30 equimolar dilute HCl with excitation wavelength of 377 nm.

**Table S1** Bond lengths for **Mo_6_-Q-NO_2_**.

| Bond | Length/Å | Bond | Length/Å | Bond | Length/Å |
| --- | --- | --- | --- | --- | --- |
| Mo1-O1 | 2.233(5) | Mo3-O1 | 2.358(5) | Mo5-O1 | 2.317(4) |
| Mo1-O7 | 1.998(5) | Mo3-O3 | 1.665(6) | Mo5-O5 | 1.674(6) |
| Mo1-O8 | 1.888(5) | Mo3-O15 | 1.965(5) | Mo5-O8 | 1.963(6) |
| Mo1-O9 | 1.885(5) | Mo3-O16 | 1.971(5) | Mo5-O12 | 1.999(7) |
| Mo1-O10 | 2.004(5) | Mo3-O17 | 1.882(5) | Mo5-O13 | 1.876(7) |
| **Mo1-N1** | **1.730(6)** | Mo3-O18 | 1.884(6) | Mo5-O15 | 1.874(6) |
| Mo2-O1 | 2.341(5) | Mo4-O1 | 2.333(5) | Mo6-O1 | 2.325(4) |
| Mo2-O2 | 1.695(6) | Mo4-O4 | 1.679(6) | Mo6-O6 | 1.695(5) |
| Mo2-O7 | 1.869(5) | Mo4-O9 | 1.955(5) | Mo6-O10 | 1.865(5) |
| Mo2-O11 | 1.987(5) | Mo4-O13 | 1.990(6) | Mo6-O11 | 1.886(5) |
| Mo2-O12 | 1.871(6) | Mo4-O14 | 1.884(5) | Mo6-O14 | 1.978(5) |
| Mo2-O18 | 1.963(6) | Mo4-O16 | 1.897(5) | Mo6-O17 | 1.974(5) |
| C1-C2 | 1.403(12) | C3-C4 | 1.415(14) | C6-C7 | 1.423(12) |
| C1-C6 | 1.448(12) | C4-C5 | 1.430(13) | C7-C8 | 1.280(14) |
| C2-C3 | 1.330(13) | C5-C6 | 1.403(13) | C8-C9 | 1.437(16) |
| O19-N3 | 1.254(10) | O20-N3 | 1.210(9) | **N1-C1** | **1.368(11)** |
| N2-C5 | 1.348(12) | N2-C9 | 1.337(13) | N3-C2 | 1.466(12) |

**Table S2** Bond Angles for **Mo_6_-Q-NO_2_**.

| Bond Angle/˚ | | Bond Angle/˚ | | Bond Angle/˚ | |
| --- | --- | --- | --- | --- | --- |
| O7 Mo1 O1 | 77.1(2) | Mo1 O1 Mo4 | 90.40(16) | O7 Mo1 O10 | 83.4(2) |
| Mo1 O1 Mo5 | 90.77(17) | O8 Mo1 O1 | 79.0(2) | Mo1 O1 Mo6 | 91.31(16) |
| O8 Mo1 O7 | 88.0(3) | Mo2 O1 Mo3 | 89.30(16) | O8 Mo1 O10 | 155.5(2) |
| Mo4 O1 Mo2 | 178.7(2) | O9 Mo1 O1 | 79.2(2) | Mo4 O1 Mo3 | 89.46(18) |
| O9 Mo1 O7 | 155.9(2) | Mo5 O1 Mo2 | 90.18(16) | O9 Mo1 O8 | 91.2(3) |
| Mo5 O1 Mo3 | 88.99(15) | O9 Mo1 O10 | 87.5(2) | Mo5 O1 Mo4 | 90.06(16) |
| O10 Mo1 O1 | 76.75(17) | Mo5 O1 Mo6 | 177.9(2) | N1 Mo1 O1 | 175.7(2) |
| Mo6 O1 Mo2 | 89.75(16) | N1 Mo1 O7 | 100.1(3) | Mo6 O1 Mo3 | 88.93(16) |
| N1 Mo1 O8 | 104.3(3) | Mo6 O1 Mo4 | 89.96(16) | N1 Mo1 O9 | 103.4(3) |
| Mo2 O7 Mo1 | 114.8(3) | N1 Mo1 O10 | 99.8(3) | Mo1 O8 Mo5 | 114.5(3) |
| O2 Mo2 O1 | 177.2(3) | Mo1 O9 Mo4 | 115.1(2) | O2 Mo2 O7 | 104.3(3) |
| Mo6 O10 Mo1 | 114.8(2) | O2 Mo2 O11 | 101.8(3) | Mo6 O11 Mo2 | 116.4(2) |
| O2 Mo2 O12 | 105.1(3) | Mo2 O12 Mo5 | 116.9(3) | O2 Mo2 O18 | 102.8(3) |
| Mo5 O13 Mo4 | 116.6(3) | O7 Mo2 O1 | 76.9(2) | Mo4 O14 Mo6 | 117.0(2) |
| O7 Mo2 O11 | 86.9(2) | Mo5 O15 Mo3 | 117.1(3) | O7 Mo2 O12 | 91.9(3) |
| Mo4 O16 Mo3 | 117.2(3) | O7 Mo2 O18 | 152.2(2) | Mo3 O17 Mo6 | 116.6(2) |
| O11 Mo2 O1 | 75.67(18) | Mo3 O18 Mo2 | 118.2(3) | O12 Mo2 O1 | 77.3(2) |
| **C1 N1 Mo1** | **167.1(6)** | O12 Mo2 O11 | 152.5(3) | O12 Mo2 O18 | 86.6(3) |
| O18 Mo2 O1 | 75.7(2) | O18 Mo2 O11 | 82.0(2) | O3 Mo3 O1 | 177.8(2) |
| O3 Mo3 O15 | 103.4(3) | O3 Mo3 O16 | 102.4(3) | O3 Mo3 O17 | 103.4(2) |
| O3 Mo3 O18 | 105.2(3) | O15 Mo3 O1 | 75.59(19) | O15 Mo3 O16 | 84.1(2) |
| O16 Mo3 O1 | 75.6(2) | O17 Mo3 O1 | 77.49(18) | O17 Mo3 O15 | 152.9(2) |
| O17 Mo3 O16 | 86.0(2) | O17 Mo3 O18 | 90.5(2) | O18 Mo3 O1 | 76.7(2) |
| O18 Mo3 O15 | 86.7(3) | O18 Mo3 O16 | 152.2(2) | O4 Mo4 O1 | 177.8(2) |
| O4 Mo4 O9 | 103.7(2) | O4 Mo4 O13 | 102.7(3) | O4 Mo4 O14 | 104.7(3) |
| O4 Mo4 O16 | 103.3(3) | O9 Mo4 O1 | 75.35(18) | O9 Mo4 O13 | 83.1(2) |
| O13 Mo4 O1 | 75.3(2) | O14 Mo4 O1 | 77.29(18) | O14 Mo4 O9 | 87.8(2) |
| O14 Mo4 O13 | 152.5(2) | O14 Mo4 O16 | 90.3(2) | O16 Mo4 O1 | 77.5(2) |
| O16 Mo4 O9 | 152.5(2) | O16 Mo4 O13 | 86.1(2) | O5 Mo5 O1 | 176.3(4) |
| O5 Mo5 O8 | 102.3(3) | O5 Mo5 O12 | 101.3(4) | O5 Mo5 O13 | 105.2(4) |
| O5 Mo5 O15 | 103.7(3) | O8 Mo5 O1 | 75.45(19) | O8 Mo5 O12 | 83.4(3) |
| O12 Mo5 O1 | 75.6(2) | O13 Mo5 O1 | 77.75(19) | O13 Mo5 O8 | 87.3(2) |
| O13 Mo5 O12 | 153.2(2) | O15 Mo5 O1 | 78.28(19) | O15 Mo5 O8 | 153.4(2) |
| O15 Mo5 O12 | 86.0(3) | O15 Mo5 O13 | 91.4(3) | O6 Mo6 O1 | 176.9(2) |
| O6 Mo6 O10 | 104.3(2) | O6 Mo6 O11 | 104.8(3) | O6 Mo6 O14 | 101.5(2) |
| O6 Mo6 O17 | 101.7(2) | O10 Mo6 O1 | 77.10(18) | O10 Mo6 O11 | 91.9(2) |
| O10 Mo6 O14 | 85.9(2) | O10 Mo6 O17 | 153.3(2) | O11 Mo6 O1 | 77.89(19) |
| O11 Mo6 O14 | 153.4(2) | O11 Mo6 O17 | 87.2(2) | O14 Mo6 O1 | 75.76(19) |
| O17 Mo6 O1 | 76.61(18) | O17 Mo6 O14 | 83.1(2) | Mo1 O1 Mo2 | 90.84(18) |
| Mo1 O1 Mo3 | 179.7(2) | C9 N2 C5 | 117.2(11) | O19 N3 C2 | 118.6(8) |
| O20 N3 C2 | 118.3(9) | O20 N3 O19 | 123.2(9) | N1 C1 C2 | 124.0(8) |
| N1 C1 C6 | 119.8(8) | C2 C1 C6 | 116.0(9) | C1 C2 N3 | 118.8(9) |
| C3 C2 N3 | 116.6(8) | C3 C2 C1 | 124.6(9) | C2 C3 C4 | 120.6(9) |
| C3 C4 C5 | 118.4(10) | N2 C5 C4 | 117.1(10) | N2 C5 C6 | 123.1(10) |
| C6 C5 C4 | 119.8(10) | C5 C6 C1 | 120.5(9) | C5 C6 C7 | 116.1(9) |
| C7 C6 C1 | 123.4(10) | C8 C7 C6 | 122.3(12) | C7 C8 C9 | 118.5(11) |
| N2 C9 C8 | 122.8(11) |  |  |  |  |
