# Supplementary material for: Reversible proton-switchable fluorescence controlled by conjugation effect in an organically-functionalized polyoxometalate
Source: Sci Rep. 2016 Jun 20;6:27861. doi: 10.1038/srep27861 (PMC4913239; doi:10.1038/srep27861)

checkCIF/PLATON report

You have not supplied any structure factors. As a result the full set of tests cannot be run.

THIS REPORT IS FOR GUIDANCE ONLY. IF USED AS PART OF A REVIEW PROCEDURE FOR PUBLICATION, IT SHOULD NOT REPLACE THE EXPERTISE OF AN EXPERIENCED CRYSTALLOGRAPHIC REFEREE.

No syntax errors found. CIF dictionary Interpreting this report

Datablock: test

```
Bond precision:  C-C = 0.0147 A          Wavelength=0.71073

Cell:          a=12.6462(8)       b=12.7095(8)      c=19.779(2)
               alpha=101.371(7)   beta=102.654(7)   gamma=101.842(6)
Temperature:   103 K
```

	Calculated	Reported
Volume	2936.6(4)	2936.6(4)
Space group	P -1	P-1
Hall group	-P 1	?
Moiety formula	2(C9 H5 Mo6 N3 O20), 4(C16 H36 N), C2 H3 N, 2(O)	?
Sum formula	C84 H157 Mo12 N11 O42	C42 H80.50 Mo6 N5.50 O21
Mr	3144.49	1574.26
Dx,g cm-3	1.778	1.780
Z	1	2
Mu (mm-1)	1.316	1.316
F000	1578.0	1582.0
F000’	1557.93	
h,k,lmax	15,15,24	15,15,24
Nref	11549	11522
Tmin,Tmax	0.523,0.900	0.559,0.902
Tmin’	0.513	

```
Correction method= MULTI-SCAN

Data completeness= 0.998          Theta(max)= 26.000

R(reflections)= 0.0647( 8133)     wR2(reflections)= 0.1461( 11522)

S = 1.068                  Npar= 754
```

The following ALERTS were generated. Each ALERT has the format
test-name_ALERT_alert-type_alert-level.
Click on the hyperlinks for more details of the test.

Alert level C

```
ABSTY02_ALERT_1_C  An _exptl_absorpt_correction_type has been given without
            a literature citation. This should be contained in the
            _exptl_absorpt_process_details field.
            Absorption correction given as multi-scan
PLAT041_ALERT_1_C Calc. and Reported SumFormula    Strings  Differ     Please Check
PLAT043_ALERT_1_C Calculated and Reported Mol. Weight Differ by ..       4.03 Check
PLAT068_ALERT_1_C Reported F000 Differs from Calcd (or Missing)...     Please Check
PLAT213_ALERT_2_C Atom O5                has ADP max/min Ratio .....      3.7 prolat
PLAT220_ALERT_2_C Large Non-Solvent  O     Ueq(max)/Ueq(min) Range        3.4 Ratio
PLAT220_ALERT_2_C Large Non-Solvent  C     Ueq(max)/Ueq(min) Range        3.1 Ratio
PLAT234_ALERT_4_C Large Hirshfeld Difference N1     --  C1      ..       0.17 Ang.
PLAT234_ALERT_4_C Large Hirshfeld Difference C113   --  C115    ..       0.23 Ang.
PLAT241_ALERT_2_C High      Ueq as Compared to Neighbors for .....       C223 Check
PLAT241_ALERT_2_C High      Ueq as Compared to Neighbors for .....       C241 Check
PLAT241_ALERT_2_C High      Ueq as Compared to Neighbors for .....       C113 Check
PLAT242_ALERT_2_C Low       Ueq as Compared to Neighbors for .....       C233 Check
PLAT250_ALERT_2_C Large U3/U1 Ratio for Average U(i,j) Tensor ....        2.4 Note
PLAT250_ALERT_2_C Large U3/U1 Ratio for Average U(i,j) Tensor ....        2.2 Note
PLAT250_ALERT_2_C Large U3/U1 Ratio for Average U(i,j) Tensor ....        2.3 Note
PLAT342_ALERT_3_C Low Bond Precision on  C-C Bonds ...............     0.0147 Ang.
PLAT413_ALERT_2_C Short Inter XH3 .. XHn     H11Q   ..  H12D    ..       2.02 Ang.
```

Alert level G

```
FORMU01_ALERT_2_G  There is a discrepancy between the atom counts in the
            _chemical_formula_sum and the formula from the _atom_site* data.
            Atom count from _chemical_formula_sum:C42 H80.5 Mo6 N5.5 O21
            Atom count from the _atom_site data:  C42 H78.5 Mo6 N5.5 O21
CELLZ01_ALERT_1_G Difference between formula and atom_site contents detected.
CELLZ01_ALERT_1_G WARNING: H atoms missing from atom site list. Is this intentional?
           From the CIF: _cell_formula_units_Z    2
           From the CIF: _chemical_formula_sum  C42 H80.50 Mo6 N5.50 O21
           TEST: Compare cell contents of formula and atom_site data

           atom    Z*formula  cif sites diff
           C         84.00     84.00    0.00
           H        161.00    157.00    4.00
           Mo        12.00     12.00    0.00
           N         11.00     11.00    0.00
           O         42.00     42.00    0.00
PLAT002_ALERT_2_G Number of Distance or Angle Restraints on AtSite         11 Note
PLAT003_ALERT_2_G Number of Uiso or Uij Restrained non-H Atoms ...          7 Report
PLAT005_ALERT_5_G No _iucr_refine_instructions_details  in the CIF     Please Do !
PLAT045_ALERT_1_G Calculated and Reported Z Differ by ...........       0.50 Ratio
PLAT083_ALERT_2_G SHELXL Second Parameter in WGHT Unusually Large.      21.06 Report
PLAT093_ALERT_1_G No su's on H-positions, refinement reported as .      mixed
PLAT232_ALERT_2_G Hirshfeld Test Diff (M-X)  Mo3    --  O3      ..        6.0 su
PLAT301_ALERT_3_G Main Residue  Disorder ........... Percentage =          7 Note
PLAT302_ALERT_4_G Anion/Solvent Disorder ........... Percentage =        100 Note
PLAT311_ALERT_2_G Isolated Disordered Oxygen Atom (No H's ?) .....        Ow1 Check
PLAT311_ALERT_2_G Isolated Disordered Oxygen Atom (No H's ?) .....        Ow2 Check
PLAT432_ALERT_2_G Short Inter X...Y Contact  O5     ..  C114    ..       3.00 Ang.
PLAT432_ALERT_2_G Short Inter X...Y Contact  N2     ..  C11     ..       2.82 Ang.
PLAT432_ALERT_2_G Short Inter X...Y Contact  C9     ..  C11     ..       2.91 Ang.
PLAT710_ALERT_4_G Delete 1-2-3 or 2-3-4 Linear Torsion Angle ... #          1 Do !
            N1  -MO1 -O1  -MO5  -144.00  3.00   1.555   1.555   1.555   1.555
PLAT710_ALERT_4_G Delete 1-2-3 or 2-3-4 Linear Torsion Angle ... #          6 Do !
            N1  -MO1 -O1  -MO6    36.00  3.00   1.555   1.555   1.555   1.555
PLAT710_ALERT_4_G Delete 1-2-3 or 2-3-4 Linear Torsion Angle ... #         11 Do !
            N1  -MO1 -O1  -MO4   126.00  3.00   1.555   1.555   1.555   1.555
```

```
PLAT710_ALERT_4_G Delete 1-2-3 or 2-3-4 Linear Torsion Angle ... #        16 Do !
            N1  -MO1 -O1  -MO2   -54.00  3.00   1.555   1.555   1.555   1.555
PLAT710_ALERT_4_G Delete 1-2-3 or 2-3-4 Linear Torsion Angle ... #        21 Do !
            N1  -MO1 -O1  -MO3    17.00  0.00   1.555   1.555   1.555   1.555
PLAT710_ALERT_4_G Delete 1-2-3 or 2-3-4 Linear Torsion Angle ... #        22 Do !
            O9  -MO1 -O1  -MO3    60.00 64.00   1.555   1.555   1.555   1.555
PLAT710_ALERT_4_G Delete 1-2-3 or 2-3-4 Linear Torsion Angle ... #        23 Do !
            O8  -MO1 -O1  -MO3   -33.00 64.00   1.555   1.555   1.555   1.555
PLAT710_ALERT_4_G Delete 1-2-3 or 2-3-4 Linear Torsion Angle ... #        24 Do !
            O7  -MO1 -O1  -MO3  -124.00 64.00   1.555   1.555   1.555   1.555
PLAT710_ALERT_4_G Delete 1-2-3 or 2-3-4 Linear Torsion Angle ... #        25 Do !
            O10 -MO1 -O1  -MO3    15.00  0.00   1.555   1.555   1.555   1.555
PLAT710_ALERT_4_G Delete 1-2-3 or 2-3-4 Linear Torsion Angle ... #        26 Do !
            O5  -MO5 -O1  -MO1    57.00  4.00   1.555   1.555   1.555   1.555
PLAT710_ALERT_4_G Delete 1-2-3 or 2-3-4 Linear Torsion Angle ... #        31 Do !
            O5  -MO5 -O1  -MO6  -122.00  7.00   1.555   1.555   1.555   1.555
PLAT710_ALERT_4_G Delete 1-2-3 or 2-3-4 Linear Torsion Angle ... #        32 Do !
            O15 -MO5 -O1  -MO6     0.00  7.00   1.555   1.555   1.555   1.555
PLAT710_ALERT_4_G Delete 1-2-3 or 2-3-4 Linear Torsion Angle ... #        33 Do !
            O13 -MO5 -O1  -MO6    94.00  7.00   1.555   1.555   1.555   1.555
PLAT710_ALERT_4_G Delete 1-2-3 or 2-3-4 Linear Torsion Angle ... #        34 Do !
            O8  -MO5 -O1  -MO6  -175.00  7.00   1.555   1.555   1.555   1.555
PLAT710_ALERT_4_G Delete 1-2-3 or 2-3-4 Linear Torsion Angle ... #        35 Do !
            O12 -MO5 -O1  -MO6   -88.00  7.00   1.555   1.555   1.555   1.555
PLAT710_ALERT_4_G Delete 1-2-3 or 2-3-4 Linear Torsion Angle ... #        36 Do !
            O5  -MO5 -O1  -MO4   148.00  4.00   1.555   1.555   1.555   1.555
PLAT710_ALERT_4_G Delete 1-2-3 or 2-3-4 Linear Torsion Angle ... #        41 Do !
            O5  -MO5 -O1  -MO2   -34.00  4.00   1.555   1.555   1.555   1.555
PLAT710_ALERT_4_G Delete 1-2-3 or 2-3-4 Linear Torsion Angle ... #        46 Do !
            O5  -MO5 -O1  -MO3  -123.00  4.00   1.555   1.555   1.555   1.555
PLAT710_ALERT_4_G Delete 1-2-3 or 2-3-4 Linear Torsion Angle ... #        51 Do !
            O6  -MO6 -O1  -MO1   118.00  4.00   1.555   1.555   1.555   1.555
PLAT710_ALERT_4_G Delete 1-2-3 or 2-3-4 Linear Torsion Angle ... #        56 Do !
            O6  -MO6 -O1  -MO5   -64.00  9.00   1.555   1.555   1.555   1.555
PLAT710_ALERT_4_G Delete 1-2-3 or 2-3-4 Linear Torsion Angle ... #        57 Do !
            O10 -MO6 -O1  -MO5    18.00  0.00   1.555   1.555   1.555   1.555
PLAT710_ALERT_4_G Delete 1-2-3 or 2-3-4 Linear Torsion Angle ... #        58 Do !
            O11 -MO6 -O1  -MO5    85.00  7.00   1.555   1.555   1.555   1.555
PLAT710_ALERT_4_G Delete 1-2-3 or 2-3-4 Linear Torsion Angle ... #        59 Do !
            O17 -MO6 -O1  -MO5    -5.00  7.00   1.555   1.555   1.555   1.555
PLAT710_ALERT_4_G Delete 1-2-3 or 2-3-4 Linear Torsion Angle ... #        60 Do !
            O14 -MO6 -O1  -MO5   -92.00  7.00   1.555   1.555   1.555   1.555
PLAT710_ALERT_4_G Delete 1-2-3 or 2-3-4 Linear Torsion Angle ... #        61 Do !
            O6  -MO6 -O1  -MO4    27.00  4.00   1.555   1.555   1.555   1.555
PLAT710_ALERT_4_G Delete 1-2-3 or 2-3-4 Linear Torsion Angle ... #        66 Do !
            O6  -MO6 -O1  -MO2  -152.00  4.00   1.555   1.555   1.555   1.555
PLAT710_ALERT_4_G Delete 1-2-3 or 2-3-4 Linear Torsion Angle ... #        71 Do !
            O6  -MO6 -O1  -MO3   -62.00  4.00   1.555   1.555   1.555   1.555
PLAT710_ALERT_4_G Delete 1-2-3 or 2-3-4 Linear Torsion Angle ... #        76 Do !
            O4  -MO4 -O1  -MO1    64.00  6.00   1.555   1.555   1.555   1.555
PLAT710_ALERT_4_G Delete 1-2-3 or 2-3-4 Linear Torsion Angle ... #        81 Do !
            O4  -MO4 -O1  -MO5   -27.00  6.00   1.555   1.555   1.555   1.555
PLAT710_ALERT_4_G Delete 1-2-3 or 2-3-4 Linear Torsion Angle ... #        86 Do !
            O4  -MO4 -O1  -MO6   155.00  6.00   1.555   1.555   1.555   1.555
PLAT710_ALERT_4_G Delete 1-2-3 or 2-3-4 Linear Torsion Angle ... #        91 Do !
            O4  -MO4 -O1  -MO2  -128.00 10.00   1.555   1.555   1.555   1.555
PLAT710_ALERT_4_G Delete 1-2-3 or 2-3-4 Linear Torsion Angle ... #        92 Do !
            O14 -MO4 -O1  -MO2    78.00 10.00   1.555   1.555   1.555   1.555
PLAT710_ALERT_4_G Delete 1-2-3 or 2-3-4 Linear Torsion Angle ... #        93 Do !
            O16 -MO4 -O1  -MO2   -16.00 10.00   1.555   1.555   1.555   1.555
PLAT710_ALERT_4_G Delete 1-2-3 or 2-3-4 Linear Torsion Angle ... #        94 Do !
            O9  -MO4 -O1  -MO2   169.00 10.00   1.555   1.555   1.555   1.555
```

```
PLAT710_ALERT_4_G Delete 1-2-3 or 2-3-4 Linear Torsion Angle ... #        95 Do !
            O13 -MO4 -O1  -MO2  -105.00 10.00   1.555   1.555   1.555   1.555
PLAT710_ALERT_4_G Delete 1-2-3 or 2-3-4 Linear Torsion Angle ... #        96 Do !
            O4  -MO4 -O1  -MO3  -116.00  6.00   1.555   1.555   1.555   1.555
PLAT710_ALERT_4_G Delete 1-2-3 or 2-3-4 Linear Torsion Angle ... #       101 Do !
            O2  -MO2 -O1  -MO1   120.00  6.00   1.555   1.555   1.555   1.555
PLAT710_ALERT_4_G Delete 1-2-3 or 2-3-4 Linear Torsion Angle ... #       106 Do !
            O2  -MO2 -O1  -MO5  -149.00  6.00   1.555   1.555   1.555   1.555
PLAT710_ALERT_4_G Delete 1-2-3 or 2-3-4 Linear Torsion Angle ... #       111 Do !
            O2  -MO2 -O1  -MO6    29.00  6.00   1.555   1.555   1.555   1.555
PLAT710_ALERT_4_G Delete 1-2-3 or 2-3-4 Linear Torsion Angle ... #       116 Do !
            O2  -MO2 -O1  -MO4   -48.00 13.00   1.555   1.555   1.555   1.555
PLAT710_ALERT_4_G Delete 1-2-3 or 2-3-4 Linear Torsion Angle ... #       117 Do !
            O7  -MO2 -O1  -MO4  -164.00 10.00   1.555   1.555   1.555   1.555
PLAT710_ALERT_4_G Delete 1-2-3 or 2-3-4 Linear Torsion Angle ... #       118 Do !
            O12 -MO2 -O1  -MO4   101.00 10.00   1.555   1.555   1.555   1.555
PLAT710_ALERT_4_G Delete 1-2-3 or 2-3-4 Linear Torsion Angle ... #       119 Do !
            O18 -MO2 -O1  -MO4    12.00 10.00   1.555   1.555   1.555   1.555
PLAT710_ALERT_4_G Delete 1-2-3 or 2-3-4 Linear Torsion Angle ... #       120 Do !
            O11 -MO2 -O1  -MO4   -74.00 10.00   1.555   1.555   1.555   1.555
PLAT710_ALERT_4_G Delete 1-2-3 or 2-3-4 Linear Torsion Angle ... #       121 Do !
            O2  -MO2 -O1  -MO3   -60.00  6.00   1.555   1.555   1.555   1.555
PLAT710_ALERT_4_G Delete 1-2-3 or 2-3-4 Linear Torsion Angle ... #       126 Do !
            O3  -MO3 -O1  -MO1   -32.00 67.00   1.555   1.555   1.555   1.555
PLAT710_ALERT_4_G Delete 1-2-3 or 2-3-4 Linear Torsion Angle ... #       127 Do !
            O17 -MO3 -O1  -MO1    15.00  0.00   1.555   1.555   1.555   1.555
PLAT710_ALERT_4_G Delete 1-2-3 or 2-3-4 Linear Torsion Angle ... #       128 Do !
            O18 -MO3 -O1  -MO1   120.00 64.00   1.555   1.555   1.555   1.555
PLAT710_ALERT_4_G Delete 1-2-3 or 2-3-4 Linear Torsion Angle ... #       129 Do !
            O15 -MO3 -O1  -MO1    30.00 64.00   1.555   1.555   1.555   1.555
PLAT710_ALERT_4_G Delete 1-2-3 or 2-3-4 Linear Torsion Angle ... #       130 Do !
            O16 -MO3 -O1  -MO1   -57.00 64.00   1.555   1.555   1.555   1.555
PLAT710_ALERT_4_G Delete 1-2-3 or 2-3-4 Linear Torsion Angle ... #       131 Do !
            O3  -MO3 -O1  -MO5   -61.00  6.00   1.555   1.555   1.555   1.555
PLAT710_ALERT_4_G Delete 1-2-3 or 2-3-4 Linear Torsion Angle ... #       136 Do !
            O3  -MO3 -O1  -MO6   119.00  6.00   1.555   1.555   1.555   1.555
PLAT710_ALERT_4_G Delete 1-2-3 or 2-3-4 Linear Torsion Angle ... #       141 Do !
            O3  -MO3 -O1  -MO4    29.00  6.00   1.555   1.555   1.555   1.555
PLAT710_ALERT_4_G Delete 1-2-3 or 2-3-4 Linear Torsion Angle ... #       146 Do !
            O3  -MO3 -O1  -MO2  -151.00  6.00   1.555   1.555   1.555   1.555
PLAT710_ALERT_4_G Delete 1-2-3 or 2-3-4 Linear Torsion Angle ... #       275 Do !
            O1  -MO1 -N1  -C1     -4.00  5.00   1.555   1.555   1.555   1.555
PLAT720_ALERT_4_G Number of Unusual/Non-Standard Labels ..........         2 Note
PLAT779_ALERT_4_G Suspect or Irrelevant (Bond) Angle in CIF .... #       178 Check
             C114 -C113 -H11E    1.555   1.555   1.555             40.10 Deg.
PLAT779_ALERT_4_G Suspect or Irrelevant (Bond) Angle in CIF .... #       188 Check
             H11F -C113 -H11G    1.555   1.555   1.555             32.40 Deg.
PLAT779_ALERT_4_G Suspect or Irrelevant (Bond) Angle in CIF .... #       189 Check
             C115 -C113 -H11H    1.555   1.555   1.555             41.70 Deg.
PLAT779_ALERT_4_G Suspect or Irrelevant (Bond) Angle in CIF .... #       324 Check
             C225 -C223 -H22D    1.555   1.555   1.555             21.70 Deg.
PLAT779_ALERT_4_G Suspect or Irrelevant (Bond) Angle in CIF .... #       329 Check
             H22C -C223 -H22K    1.555   1.555   1.555             16.30 Deg.
PLAT779_ALERT_4_G Suspect or Irrelevant (Bond) Angle in CIF .... #       331 Check
             C224 -C223 -H22J    1.555   1.555   1.555             17.80 Deg.
PLAT779_ALERT_4_G Suspect or Irrelevant (Bond) Angle in CIF .... #       372 Check
             C245 -C241 -C242    1.555   1.555   1.555             30.30 Deg.
PLAT779_ALERT_4_G Suspect or Irrelevant (Bond) Angle in CIF .... #       384 Check
             H24B -C241 -H24C    1.555   1.555   1.555             29.20 Deg.
PLAT779_ALERT_4_G Suspect or Irrelevant (Bond) Angle in CIF .... #       388 Check
             H24A -C241 -H24D    1.555   1.555   1.555             32.20 Deg.
PLAT790_ALERT_4_G Centre of Gravity not Within Unit Cell: Resd.  #         3 Note
```

```
              C16 H36 N
PLAT860_ALERT_3_G Number of Least-Squares Restraints .............         90 Note
```

```
  0 ALERT level A = Most likely a serious problem - resolve or explain
  0 ALERT level B = A potentially serious problem, consider carefully
 18 ALERT level C = Check. Ensure it is not caused by an omission or oversight
 84 ALERT level G = General information/check it is not something unexpected

  8 ALERT type 1 CIF construction/syntax error, inconsistent or missing data
 21 ALERT type 2 Indicator that the structure model may be wrong or deficient
  3 ALERT type 3 Indicator that the structure quality may be low
 69 ALERT type 4 Improvement, methodology, query or suggestion
  1 ALERT type 5 Informative message, check
```

It is advisable to attempt to resolve as many as possible of the alerts in all categories. Often the minor alerts point to easily fixed oversights, errors and omissions in your CIF or refinement strategy, so attention to these fine details can be worthwhile. In order to resolve some of the more serious problems it may be necessary to carry out additional measurements or structure refinements. However, the purpose of your study may justify the reported deviations and the more serious of these should normally be commented upon in the discussion or experimental section of a paper or in the "special_details" fields of the CIF. checkCIF was carefully designed to identify outliers and unusual parameters, but every test has its limitations and alerts that are not important in a particular case may appear. Conversely, the absence of alerts does not guarantee there are no aspects of the results needing attention. It is up to the individual to critically assess their own results and, if necessary, seek expert advice.

Publication of your CIF in IUCr journals

A basic structural check has been run on your CIF. These basic checks will be run on all CIFs submitted for publication in IUCr journals (*Acta Crystallographica*, *Journal of Applied Crystallography*, *Journal of Synchrotron Radiation*); however, if you intend to submit to *Acta Crystallographica Section C* or *E*, you should make sure that full publication checks are run on the final version of your CIF prior to submission.

Publication of your CIF in other journals

Please refer to the *Notes for Authors* of the relevant journal for any special instructions relating to CIF submission.

PLATON version of 24/07/2014; check.def file version of 24/07/2014

Datablock test - ellipsoid plot

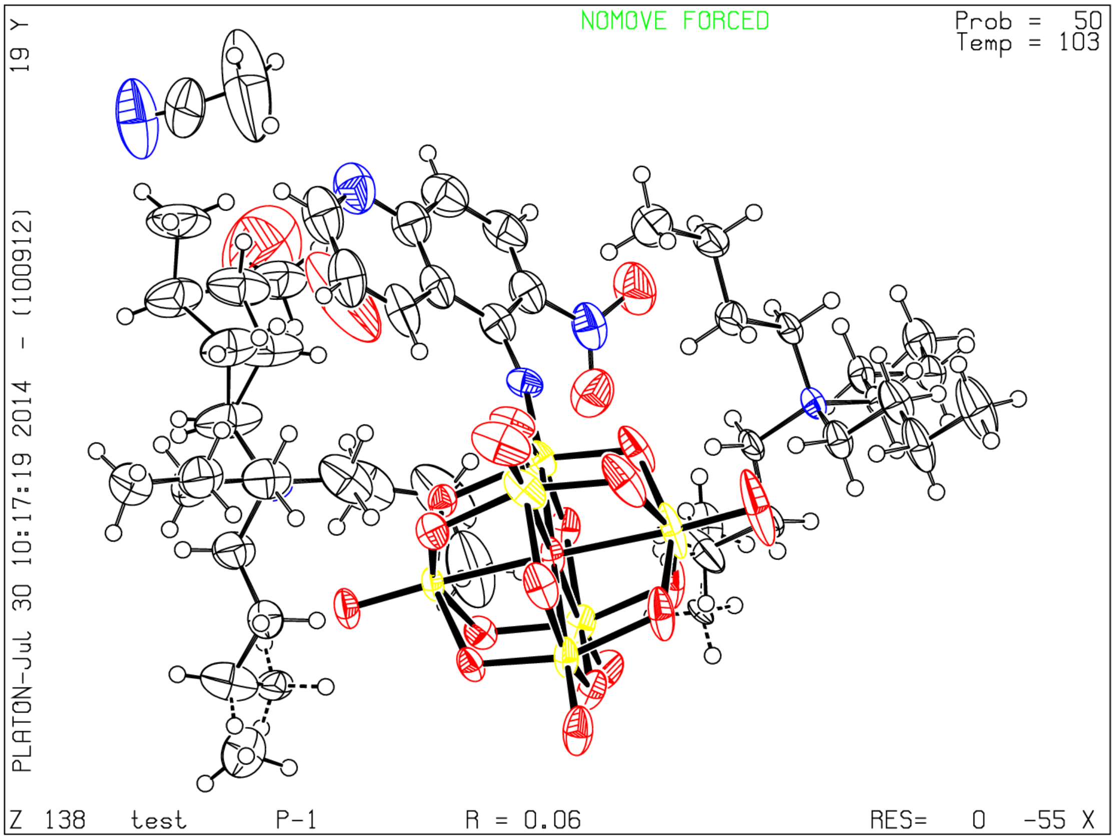

Supplement: Supplementary Dataset 2 [file srep27861-s3.zip › Dataset 2-checkcif.pdf]
